# Chemical Conversion Pathways and Kinetic Modeling for the OH-Initiated Reaction of Triclosan in Gas-Phase

**DOI:** 10.3390/ijms16048128

**Published:** 2015-04-10

**Authors:** Xue Zhang, Chenxi Zhang, Xiaomin Sun, Lingyan Kang, Yan Zhao

**Affiliations:** 1 Environment Research Institute, Shandong University, Jinan 250100, China; E-Mails: zxue_117@126.com (X.Z.); sdzhangcx@163.com (C.Z.); lingyan_k@163.com (L.K.); zhaoyan08319@126.com (Y.Z.); 2 Department of Resource and Environment, Binzhou University, Binzhou 256600, China; 3 School of Life Sciences, Qufu Normal University, Qufu 273165, China

**Keywords:** triclosan, reaction mechanism, kinetic property, formation of PCDD and PCB

## Abstract

As a widely used antimicrobial additive in daily consumption, attention has been paid to the degradation and conversion of triclosan for a long time. The quantum chemistry calculation and the canonical variational transition state theory are employed to investigate the mechanism and kinetic property. Besides addition and abstraction, oxidation pathways and further conversion pathways are also considered. The OH radicals could degrade triclosan to phenols, aldehydes, and other easily degradable substances. The conversion mechanisms of triclosan to the polychlorinated dibenzopdioxin and furan (PCDD/Fs) and polychlorinated biphenyls (PCBs) are clearly illustrated and the toxicity would be strengthened in such pathways. Single radical and diradical pathways are compared to study the conversion mechanism of dichlorodibenzo dioxin (DCDD). Furthermore, thermochemistry is discussed in detail. Kinetic property is calculated and the consequent ratio of *k*_add_/*k*_total_ and *k*_abs_/*k*_total_ at 298.15 K are 0.955 and 0.045, respectively. Thus, the OH radical addition reactions are predominant, the substitute position of OH radical on triclosan is very important to generate PCDD and furan, and biradical is also a vital intermediate to produce dioxin.

## 1. Introduction

Triclosan, (5-chloro-2-(2,4-dichlorophenoxy)-phenol, TCS), has been widely used as a broad spectrum antibacterial which is incorporated in pharmaceutical and personal care products (PPCPs) [1]. Triclosan has positive effects on the eliminating of various kinds of bacteria and fungi by entering the germs’ cell wall, and then disturbing the cell plasma and membrane composition [2,3]. Growing concern about personal health simulates the enormous use of triclosan all over the world and also a large amount of waste.

Through treated or untreated systems, triclosan effluent enters rivers, drink water, soil and dusts, accumulated in the environment [4,5,6,7]. The significant removal rate was reported to be 0.03 day^−1^ in photolysis [8]. In national sewage sludge investigation in America, triclosan was reported to be the most abundant PPCPs of all the detected samples [9]. In China, TCS has been observed in the range from 4.4 to 478 ng/L in several main rivers [10]. The TCS was removed from the European Union additive list of plastics as food-contact materials in 2010 because of its toxicity. Aquatic organisms were reported to be sensitive to the acute and chronic toxicity of triclosan. The no observed effect concentration (NOEC) of rainbow trout is 34 μg/L [11,12,13,14]. In addition, TCS shows endocrine disruption for water species [15,16,17,18]. TCS was also detected in human milk and urine samples, which could lead to carcinogenic hazard [19,20,21]. Besides the toxicity of triclosan, its conversion and formation in the environment draws more attention recently. Triclosan can be degraded through a variety of ways to generate other phenols and quinones, and various chlorinated dioxins were reported to be produced when exposed to sunlight [22,23]. Recent studies have suggested that advanced oxidation processes (AOPs) are promising to eliminate triclosan. Fenton agent with OH radical can oxidize triclosan to generate 2,4-dichlorophenol, 4-chlorocatechol, and chloro-*p*-benzoquione [24,25,26]. By means of photocatalytic method, dibenzo-*p*-dioxin and 2,8-dichlorodibenzo-*p*-dioxin (2,8-DCDD) were detected during degradation [27,28]. OH addition was proposed to initiate its degradation to two phenols [29]. However, the exact addition position on the TCS structure is uncertain. Kazushi *et al.* reported that 2,8-DCDD appeared in water samples with TCS after 3-day irradiation. The half-lives of triclosan in the freshwater and seawater last for approximately 8 and 4 days, respectively [30]. Prado *et al.* identified dichlorohydroxydibenzofuran as product in photodegradation [31]. Although the conversion to dioxin is not a dominant degradation process (0.5%–2.5%) [30], yet such an amount of transformation to DCDD is very dangerous for the environment.

Previous reports about the degradation of TCS mainly depended on experimental methods. To explain its OH initiated degradation to by-products, several degradation pathways have been proposed. However, the detailed mechanism and fate of TCS is still unclear. Thus, it is worthy to predict the precise mechanism by quantum chemistry methods. We chose the gas-phase reaction to study the basic mechanism and kinetics of TCS conversion. OH radical is chosen as the inducer which has been proved to be of great significance in the removal of various pollutants [32,33]. Physicochemical properties and kinetics are calculated to further elaborate the mechanism for dioxins formation and other products in detail.

## 2. Results and Discussion

Triclosan can be degraded by oxidants such as OH, NO_3_ and O_3_ via varied environmental after-treatment or atmospheric processes. OH radical plays a vital role in both atmospheric process and wastewater treatments due to its high oxidability. Thus, the OH-initiated reactions of triclosan are chosen to investigate degradation and conversion mechanism.

### 2.1. OH Addition Pathways

**Figure 1:**
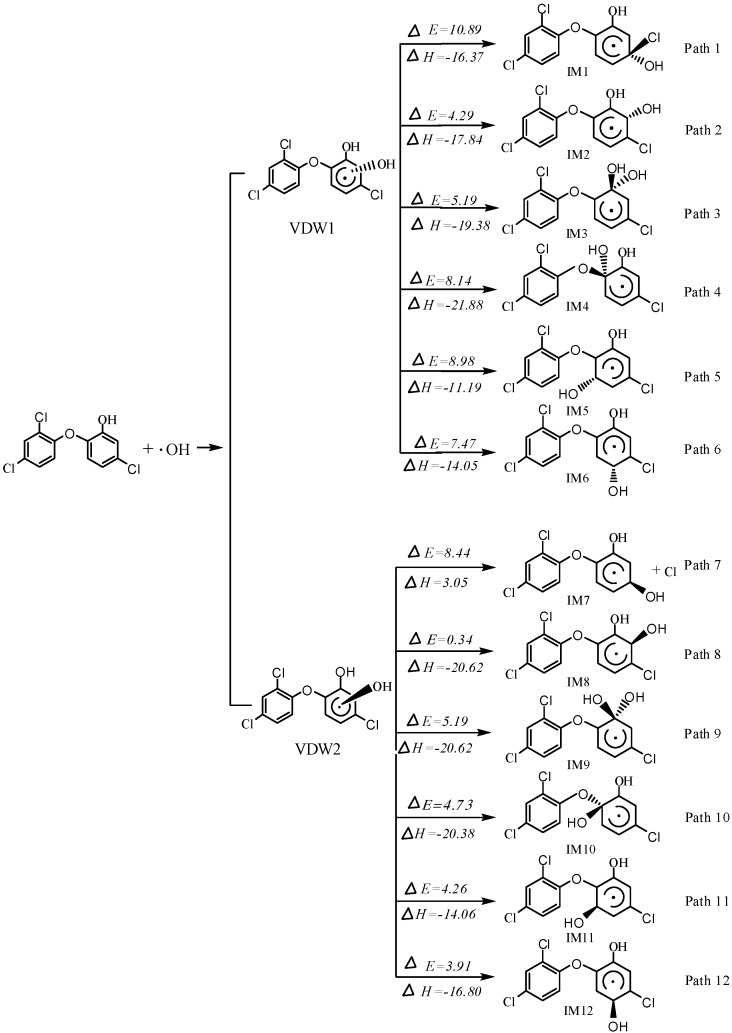

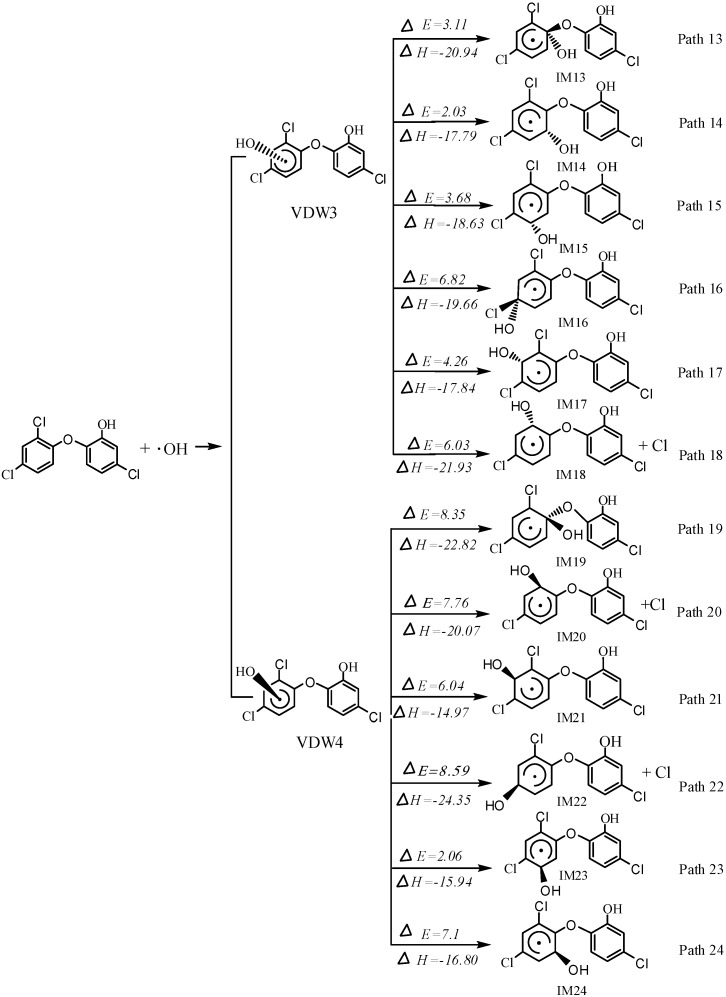
Reaction heats (ΔH) and energy barriers (ΔE) of the addition pathways (kcal·mol^−1^).

There are 24 different positions which can be attacked by OH radical in the two benzene rings. 24 positions are defined as C_1_*s/a*~C_12_*s/a* with *s* standing for *cis*-form, meaning that OH radical is on the same side relative to Cl atom, and *a* standing for anti-form which means that OH radical is on the opposite side relative to Cl atom. For convenience, the OH substituted benzene ring is defined as ring 1, and the other ring is ring 2. The energy barriers and reaction heats in primary reactions are shown in Figure 1.

Given each side of the two benzene rings, 4 van der waals complexes are found in 24 reactions to form TCS–OH adducts. Overall, there are identical reactivities to OH radical in both rings. TCS–OH isomers are formed via 24 transition states with energy barriers varying from 0.34 to 10.89 kcal·mol^−1^. The distance between OH radical and carbon inside the rings ranges from 1.961 to 2.003 Å. The lowest barrier is 0.34 kcal·mol^−1^ in IM8, *ortho*-position of OH substituent on the ring 1, releasing 20.62 kcal·mol^−1^ of energy which can initiate further reactions more easily. In addition, the most difficult addition happens to IM1. Most of the addition reactions are strongly exothermic, giving out about 20 kcal·mol^−1^ of energy, and only pathway 7 is endothermic which suggests that it is not an energy favorable pathway. The energy barriers are determined by both charge distribution and stereo hindrance. Additions are mostly more energy favorable when OH radical attacks VDW2 and VDW3 from the same side, owing to smaller stereo effects as displayed in Figure 1.

The number of Cl atoms can partly determine the toxicity of substituted TCS. IM1, IM7, IM16, IM18, IM20 and IM22 with the structure of both Cl and OH group on the same carbon atom are produced via OH addition to the Cl substituted carbon. These structures are proved to facilitate the chlorine atom elimination from the ring in our calculation. It is noted that chlorine atoms are off the rings in IM7, IM18, IM20 and IM22. Meanwhile, the C–Cl bonds and the newly formed C–O bonds in IM1 and IM16 are 1.842 and 1.395 Å, respectively. In addition, the length of the other four newly formed C–Cl bonds are stretched by nearly 1.950 Å, about 13% longer compared to the initial one, which could account for the easier Cl elimination.

**Figure 2:**
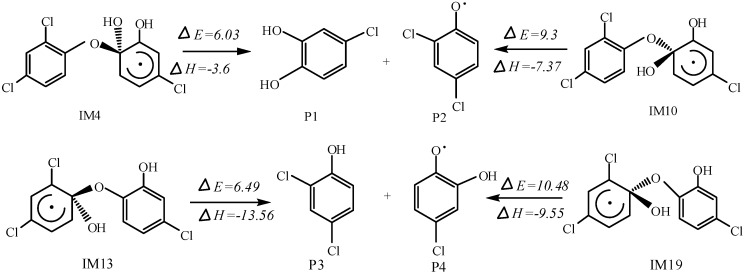
Reaction heats (ΔH) and energy barriers (ΔE) of 4 bond breaking pathways of degradation (kcal·mol^−1^).

Phenol-type intermediates have been detected as products in the OH initiated photocatalysis degradation. Stamatis *et al.* proposed a possible pathway that dichlorophenols generated from OH addition on triclosan [29]. In OH addition reaction, unbalance of electron density may lead to the triclosan breakage to two Phenols [34]. In structures of IM4, IM10, IM13 and IM 19, the O atom of OH substituent and the bridge O share the same C atom, and C–O bond may break more easily (Figure 2) These structures have already been reported to decompose to a mono-ring radical and a mono-ring molecule by our previous work [35]. The IM4 and IM10 can be decomposed to P1 and P2 with energy barriers of 6.03 and 9.30 kcal·mol^−1^, respectively. The IM13 and IM19 can be dissociated to P3 and P4 with energy barriers of 6.49 and 10.48 kcal·mol^−1^, respectively. All the reactions are exothermic, releasing the energy with 3.6–13.56 kcal·mol^−1^. P2 and P4 radicals can be converted easily in the next step. The energy barriers and reaction heats of C–O breaks are compared in the 4 position, IM4 and IM13 show energy favorable. We suggest that bond breaks after OH addition is a reasonable pathway. In addition, generation of 2,4-dichlorophenol and 4-chlorocatechol is consistent with advanced oxidation processes degradation experiment, they may degrade to phenol, till to CO_2_ and H_2_O [24,26]. This proved that OH addition is reasonable to explain triclosan degradation to two phenols. The mechanism has been given by quantum chemistry calculation, showing that addition beside the bridge O are energy favorable.

### 2.2. OH Abstraction

**Figure 3:**
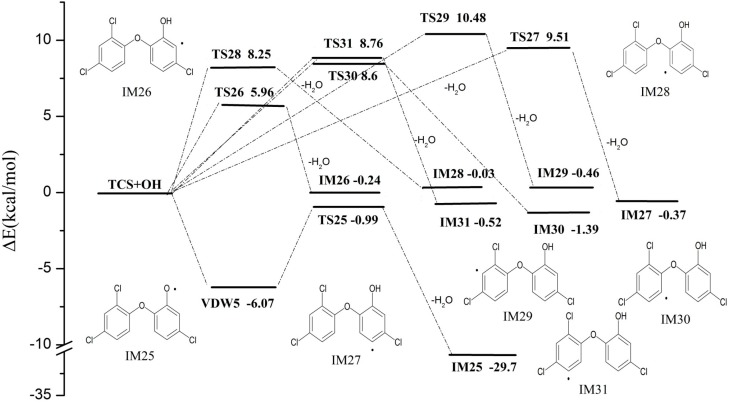
Profile of the potential energy surface for abstraction pathways (kcal·mol^−1^).

**Figure 4:**
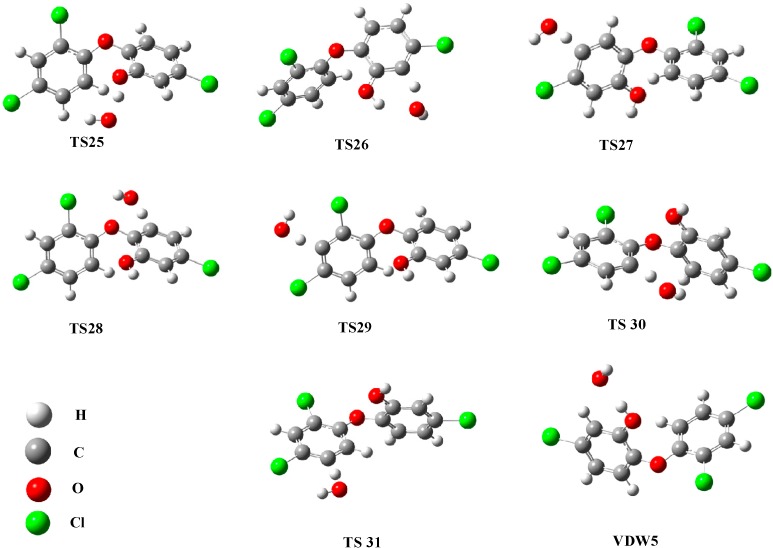
Structures of transition states of OH abstraction of triclosan.

**Figure 5:**
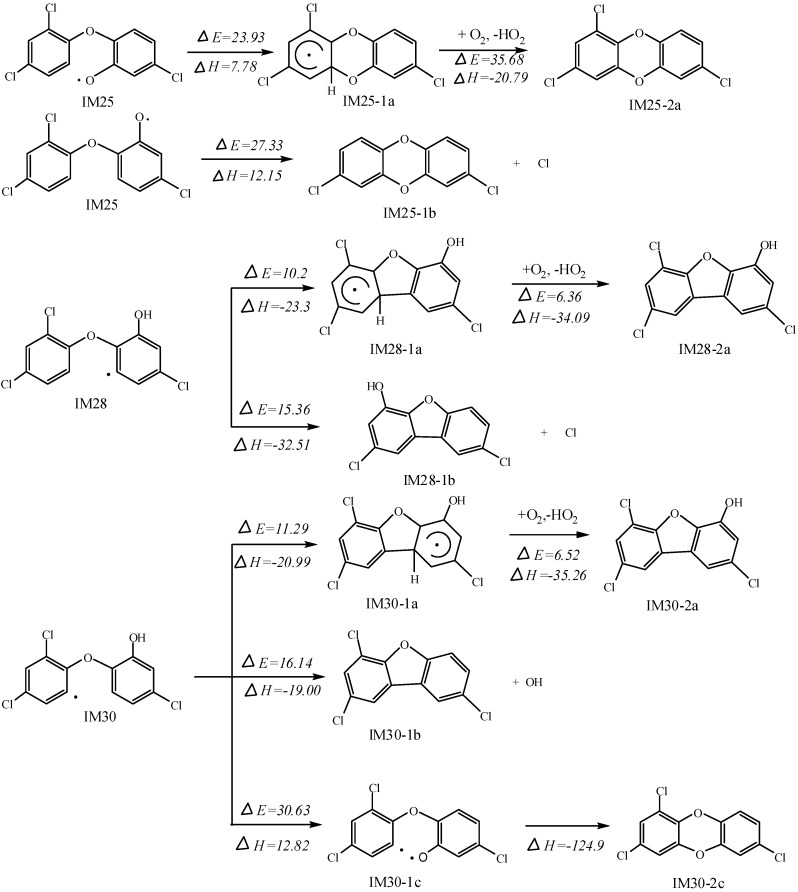
**Figure** **5.** Formation of dichlorodibenzo dioxin and furan (DCDD/Fs) pathways (kcal·mol^−1^).

TCS can also be degraded through the pathway of OH abstraction. OH radical can abstract H atom to produce water and various intermediates from 7 different positions in TCS as shown in Figure 3. It should be noted that IM25, one of the precursors of DCDD/Fs, is produced from VDW5 via TS25 (Figure 4) whose energy barrier is low (Figure 5). This pathway is completely different from other OH abstraction ones. Meanwhile, the energy barrier of this pathway is also comparable to the OH addition pathways and strongly exothermic, giving out 29.7 kcal·mol^−1^ of energy. It is obvious that this pathway is reasonable though it competes with OH addition pathways. The intermediates, IM26–IM31, are formed via TS26–TS31 with energy barriers ranging from 5.96 to 10.48 kcal·mol^−1^. These energy barriers are comparable to the reaction of OH addition, and these six pathways are only slightly exothermic, releasing energy up to 0.03–1.39 kcal·mol^−1^.

Based on the above discussion, the TCS can be degraded easily via reactions initiated by OH radical. The OH addition pathways are more energy favorable and predominating among the primary reactions. However, the OH abstraction pathways are not negligible compared to OH addition pathways especially the pathway producing IM25, which may lead to the formation of DCDD/Fs. Thus, it may be possible that TCS are potentially greatly hazardous to the environment. 

### 2.3. Formation of Polychlorinated Dibenzopdioxin and Furan (PCDD/Fs)

Given that IM25, IM28 and IM 30 were produced via OH abstraction of the OH substitution as the precursors of the formation of DCDD/Fs, they are chosen to investigate the potential to be converted to DCDD/Fs with great toxicity (Figure 5).

In IM25, the O atom generated via abstraction can attack two C atoms adjacent to the bridged O atom. The product IM25-1a can be produced in the attack towards C atom without Cl substituent. This reaction has to overcome an energy barrier of 23.93 kcal·mol^−1^ and is endothermic, discharging 7.78 kcal·mol^−1^ of energy. Then the H atom can be abstracted by O_2_ to form IM25-2a with potential energy of 35.68 kcal·mol^−1^. In addition, the other ring-closure pathway for IM25 involves the attack at the C atom with Cl substituent. This process is also endothermic, discharging 12.15 kcal·mol^−1^ of energy but there is a higher energy barrier of 27.33 kcal·mol^−1^ mainly because of stereo hindrance and a larger twist angle. Then IM25-1b (DCDD) and Cl atom are generated more easily than IM25-2a. In the case of IM30, the C atom with unpaired electron behaves like the O atom substituent in IM25, since it can attack two C atoms beside the bridged O atom. The attack at the C atom with OH substituent leads to the formation of IM30-1b and OH radical. This process is exothermic, with 19.00 kcal·mol^−1^ of energy released and similarly there is a higher energy barrier of 16.14 kcal·mol^−1^. When the C atom without OH substituent is attacked, IM30-1a will be formed with an energy barrier of 11.29 kcal·mol^−1^, and this process is exothermic, giving out energy with 20.99 kcal·mol^−1^. The O_2_ molecule will subsequently abstract the H atom in IM28-1a and IM30-1a. In these reactions, energy barriers are 6.36 and 6.52 kcal·mol^−1^, respectively. Furthermore, both reactions are strongly exothermic, giving out 34.09 and 35.26 kcal·mol^−1^, respectively.

The C atom with unpaired electrons in IM28 can attack the C atom on the C–Cl bond next to the bridged O atom. A ring-closure reaction is happened. PCDF and Cl atom are produced in this strongly exothermic (−32.51 kcal·mol^−1^) process the energy barrier is identical with that of IM30.

The intermediates produced via OH abstraction, IM25, IM28 and IM30, were investigated due to their high potential to form PCDD/Fs. In the present study the formation of PCDFs via IM28 and IM30 are more energy favorable than that of DCDDs via IM25. However, the formations of IM28 and IM30 are less energy favorable than the formation of IM25. Obviously, the TCS has the potential to be transformed to PCDD/Fs, which can be supported by kinetic studies in the following section. This result agrees with the conclusion that that DCDD and furan are by-products during degradation by previous studies [23,27]. The potential energy of these pathways shows that energy support is needed to generate dioxin and furan compounds. Photolysis is reasonable to promote the conversion efficiency. In this calculation, abstraction is very import for further reaction to produce more toxic products.

### 2.4. Formation of Polychlorinated Biphenyls (PCBs)

**Figure 6:**
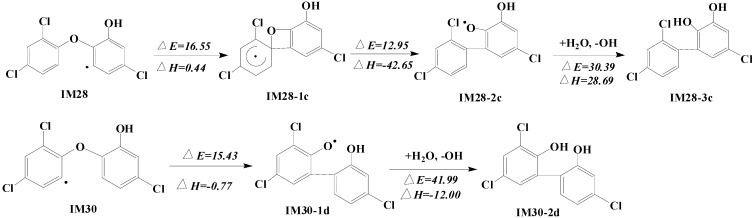
Formation of PCBs pathways (kcal·mol^−1^).

In previous photolysis research, it has been found that polychlorinated biphenyl diol (OH)_2_ PCB is also detected as a vital triclosan photolysis product [36,37]. Sarah Kliegman *et al.* suggested that biradical intermediate is a probable pathway to generate spirobenzoxetane known as precursor of DCDD and (OH)_2_ PCB [38]. To find out whether the single OH initiated reactions can be probable, theoretical calculation is arranged to investigate the mechanism of (OH)_2_ PCB formation. As shown in Figure 6, the OH abstraction products IM28 and IM30 are also likely to convert into a four-membered ring. A potential barrier of 16.55 kcal·mol^−1^ should be overcome to generate IM28-1c, and such a process turns out endothermic (0.44 kcal·mol^−1^). Thus, a 4-membered ring will be opened in the further reaction. Great heat is released (−42.65 kcal·mol^−1^) with energy barrier of 12.95 kcal·mol^−1^, which can lead to the transformation of IM28-2c, a kind of PCB with high toxicity. When IM30 becomes a reactant, one comparatively energy favorable pathway is discovered. Only one step is necessary to convert IM30 directly into IM30-1d, isomer of IM28-2c. The calculation result about transition states from IM30 to IM30-1d clearly testifies that such a four-member ring structure may be the intermediate of degradation products. However, an OH initiated single radical is possible to generate PCB. The IM28-2c and IM30-1d can produce IM28-3c and IM30-2d by abstracting the H atom from H_2_O.

The biradical formation in the process of converting into dioxin is also calculated. The IM30 can be further abstracted by O_2_ with energy barrier of 30.63 kcal·mol^−1^ and absorb 12.82 kcal·mol^−1^ of heat. Thus, the second radical is acquired on the OH substituted position. After strong heat is released, the IM30-2c is formed as a type of important DCDDs. Compared with IM25 discussed earlier, more energy is needed for IM30 to overcome the barrier and get biradical. However, in this pathway, no more energy consumption is needed to convert into IM30-2c. To get biradicals, we have also tested other pathways, but calculation showed that biradicals are impossible to produce in other positions. Biradical like IM30-1c structure is very active. It could lead to both intramolecular conversion and intermolecular reaction. In this pathway, it is possible to produce dioxin in intramolecular reaction. In addition, OH is impossible to abstract the same structure again and get two radicals. In contrast, the O_2_ is crucial for the biradical transformation. Thus, we suggest that IM25 (Figure 5) and IM30 can bring about three important pathways to produce dioxin in the environment. In addition, both single radical and biradical are probable for the reaction.

### 2.5. Kinetics

The canonical variational transition state theory (CVT) with small-curvature tunneling (SCT) correction theory is employed to carry out the kinetic calculations. The relationship between the temperature and rate constants is studied with temperature from 200 to 400 K. The rate constants for OH reaction with TCS are fitted in the Arrhenius formula *k* = A exp(−Ea/RT) as shown in Table 1. Given that we mainly focus on reactions in gas phase, only the rate constants of 298 K are illustrated. In the multichannel reaction of TCS with OH radicals, the rate constants of overall OH radical addition reaction and H atom abstraction are defined as *k*_add_ and *k*_abs_, respectively. The overall rate constant for the TCS with OH reaction is labeled as *k*_total_, in which *k*_total_ = *k*_add_ + *k*_abs_. *k*_add_/*k*_total_ and *k*_abs_/*k*_total_ are the branching ratios (R) for the whole reaction. Variations of *k*_add_, *k*_abs_, *k*_total_, *k*_add_/*k*_total_ and *k*_abs_/*k*_total_ at 200–400 K are listed in Table 1. At 298.15 K, the *k*_total_ of TCS with OH is 1.02 × 10^−13^ cm^3^·molecule^−1^·s^−1^.

At 298.15 K, *k*_add_/*k*_total_ and *k*_abs_/*k*_total_ are 0.955 and 0.045, respectively. Thus, the OH radical addition reactions are predominant over H atom abstraction of TCS at room temperature. However, the branching ratios of OH radical addition reaction and H atom abstraction reaction will change rapidly as the temperature varies. The proportion of H atom abstraction reactions will become higher as the temperature rises. Compared with the above section, the rate constant of pathway 25 is 3.22 × 10^−15^ cm^3^·molecule^−1^·s^−1^, which is comparatively high, and this path is very important when PCDD/Fs are formed.

**Table 1:** Rate constants *k* (cm^3^·molecule^−1^·s^−1^) at 298.15 K, and Arrhenius formulas for the reactions of TCS with OH radical over the temperature range of 200–400 K.

Reactions	*k*_298.15 K_	Arrhenius Formulas
TCS + OH → IM1	1.09 × 10^−18^	*k*(T) = 6.9 × 10^−14^ exp(−2902.9/T)
TCS + OH → IM2	4.19 × 10^−18^	*k*(T) = 6.9 × 10^−14^ exp(−2902.9/T)
TCS + OH → IM3	6.68 × 10^−16^	*k*(T) = 2.5 × 10^−11^ exp(−1075.8/T)
TCS + OH → IM4	8.08 × 10^−15^	*k*(T) = 3.8 × 10^−14^ exp(−454.3/T)
TCS + OH → IM5	4.84 × 10^−15^	*k*(T) = 1.3 × 10^−13^ exp(−971.5/T)
TCS + OH → IM6	4.51 × 10^−14^	*k*(T) = 6.7 × 10^−14^ exp(−106.9/T)
TCS + OH → IM7	1.40 × 10^−17^	*k*(T) = 5.0 × 10^−14^ exp(−2447.1/T)
TCS + OH → IM8	1.34 × 10^−16^	*k*(T) = 2.8 × 10^−14^ exp(1597.7/T)
TCS + OH → IM9	2.54 × 10^−17^	*k*(T) = 6.0 × 10^−14^ exp(−2321.3/T)
TCS + OH → IM10	2.60 × 10^−16^	*k*(T) = 5.8 × 10^−14^ exp(−1611.7/T)
TCS + OH → IM11	5.33 × 10^−15^	*k*(T) = 4.2 × 10^−14^ exp(−610.4/T)
TCS + OH → IM12	1.97 × 10^−14^	*k*(T) = 3.6 × 10^−14^ exp(−174.1/T)
TCS + OH → IM13	9.14 × 10^−16^	*k*(T) = 5.9 × 10^-14^ exp(−1242.2/T)
TCS + OH → IM14	7.38 × 10^−17^	*k*(T) = 3.0 × 10^−14^ exp(−1789.7/T)
TCS + OH → IM15	8.85 × 10^−15^	*k*(T) = 8.3 × 10^−14^ exp(−660.8/T)
TCS + OH → IM16	2.15 × 10^−16^	*k*(T) = 5.3 × 10^−14^ exp(−1642.9/T)
TCS + OH → IM17	2.60 × 10^−16^	*k*(T) = 2.0 × 10^−14^ exp(−1989.7/T)
TCS + OH → IM18	4.05 × 10^−16^	*k*(T) = 2.7 × 10^−14^ exp(−1254.0/T)
TCS + OH → IM19	1.20 × 10^−18^	*k*(T) = 1.9 × 10^−14^ exp(−2887.7/T)
TCS + OH → IM20	3.42 × 10^−17^	*k*(T) = 2.8 × 10^−14^ exp(−1998.8/T)
TCS + OH → IM21	1.26 × 10^−16^	*k*(T) = 9.9 × 10^−14^ exp(−1990.4/T)
TCS + OH → IM22	2.03 × 10^−16^	*k*(T) = 3.5 × 10^−14^ exp(−1535.9/T)
TCS + OH → IM23	9.77 × 10^−16^	*k*(T) = 7.0 × 10^−15^ exp(−579.5/T)
TCS + OH → IM24	1.03 × 10^−15^	*k*(T)=8.7 × 10^−15^ exp(−631.4/T)
TCS + OH → IM25 + H_2_O	3.22 × 10^−15^	*k*(T) = 6.7 × 10^−14^ exp(−699.7/T)
TCS + OH → IM26 + H_2_O	1.19 × 10^−15^	*k*(T) = 5.9 × 10^−13^ exp(−1159.1/T)
TCS + OH → IM27 + H_2_O	1.31 × 10^−19^	*k*(T) = 5.3 × 10^−13^ exp(−4558.9/T)
TCS + OH → IM28 + H_2_O	1.18 × 10^−16^	*k*(T) = 2.1 × 10^−13^ exp(−2243.9/T)
TCS + OH → IM29 + H_2_O	3.94 × 10^−19^	*k*(T) = 4.5 × 10^−13^ exp(−4176.5/T)
TCS + OH → IM30 + H_2_O	2.28 × 10^−17^	*k*(T) = 7.7 × 10^−14^ exp(−2429.8/T)
TCS + OH → IM31 + H_2_O	3.03 × 10^−17^	*k*(T) = 8.8 × 10^−13^ exp(−3077.9/T)

At 298.15 K, *k*_add_ = 9.72 × 10^−14^, *k*_abs_ = 4.58 × 10^−15^, *k*_total_ = 1.02 × 10^−13^.

## 3. Computational Methods

### 3.1. Density Functional Theory

The OH-initiated reaction was calculated by means of DFT (Density Functional Theory) with Gaussian 03 programs. MPWB1K was set to study reaction energy and barrier heights which have been proved precise for thermochemistry [39]. 6-31+G(d, p) basis sets can excellently describe the wave-function and is relatively efficient. Structures of reactants, transition states, intermediates, and products are calculated at the MPWB1K/6-31+G(d, p) level, which has been applied successfully in previous researches [40].

To verify each transition state point to connect the designated reactants and products, the intrinsic reaction coordinate (IRC) analysis was performed. A more flexible basis set, 6-311+G-(3df, 2p) was employed to calculate the single point energies.

### 3.2. Kinetic Calculation

The CVT/SCT method was used to calculate rate constants. We selected 80 points between reactants and products [41,42,43]. From each side near the transition state listed 40 points to keep the minimum energy path (MEP). POLYRATE 9.7 was employed for the calculation of chemical reaction rate.

## 4. Conclusions

OH radicals are more easily added to the aromatic ring, compared with the abstraction of H from the aromatic ring. At 298.15 K, the overall rate constant of the TCS with OH radical is about 1.02 × 10^−13^ cm^3^·molecule^−1^·s^−1^. The OH radical addition reactions are predominant over H atom abstraction in the primary reaction of TCS. As the temperature rises, the branching ratio of OH radical addition reaction will decrease, while the proportion of H atom abstraction reaction will present the opposite case.

The result of this work tests the OH initiated degradation by-products of triclosan. Probable mechanism is proposed with the quantum chemistry method. Part of the TCS–OH adducts could be decomposed to lower toxic products with respect to TCS. Furthermore, the TCS–OH radical isomers are activated radicals and can be further oxidized by oxidants such as O_2_ in the gas phase. OH abstraction is a precursor reaction to produce more toxic products. Although the *k*_abs_/*k*_total_ is low, the observation of dioxin in laboratory proved that OH abstraction is also influential. The proposed pathways show that there is significant potential in TCS to produce DCDD/Fs and PCBs with great toxicity via H abstraction reactions. The diradical pathway to form DCDD from triclosan is also important. It would be a considerable hazard if a large amount of triclosan is discharged into the environment. Because it is the precursor of these toxic compounds, even a small proportion could influence public health.

## References

[1] Furia T.E., Schenkel A.G. (1968). Recent development in methods for assessing performance of antibacterial toilet soaps. Soap Chem. Spec..

[2] Russell A.D. (2004). Whither triclosan?. J. Antimicrob. Chemother..

[3] McMurry L.M., Oethinger M., Levy S.B. (1998). Triclosan targets lipid synthesis. Nature.

[4] Perez A.L., Sylor M.A.D., Slocombe A.J., Lew M.G. (2013). Triclosan occurrence in freshwater systems in the United States (1999–2012): A meta-analysis. Environ. Toxicol. Chem..

[5] Federle T.W., Kaiser S.K., Nuck B.A. (2002). Fate and effects of triclosan in activated sludge. Environ. Toxicol. Chem..

[6] Ying G.G., Kookana R.S., Kolpin D.W. (2009). Occurrence and removal of pharmaceutically active compounds in sewage treatment plants with different technologies. J. Environ. Monit..

[7] Lozano N., Rice C.P., Ramirez M., Torrents A. (2013). Fate of triclocarban, triclosan and methyltriclosan during wastewater and biosolids treatment processes. Water Res..

[8] Singer H., Müller S.T.C., Pillonel L. (2002). Triclosan: Occurrence and fate of a widely used biocide in the aquatic environment: Field measurements in wastewater treatment plants, surface waters, and lake sediments. Environ. Sci. Technol..

[9] McClellan K., Halden R.U. (2010). Pharmaceuticals and personal care products in archived U.S. biosolids from the 2001 EPA national sewage sludge survey. Water Res..

[10] Zhao J.L., Zhang Q.Q., Chen F., Wang L., Ying G.G., Liu Y.S., Yang B., Zhou L.J., Liu S., Su H.C. (2013). Evaluation of triclosan and triclocarban at river basin scale using monitoring and modeling tools: Implications for controlling of urban domestic sewage discharge. Water Res..

[11] Orvos D.R., Versteeg D.J., Inauen J., Capdevielle M., Rothenstein A., Cunningham V. (2002). Aquatic toxicity of triclosan. Environ. Toxicol. Chem..

[12] Francesco P., Luca N. (2013). Assessing triclosan-induced ecological and *trans*-generational effects in natural phytoplankton communities: A trait-based field method. Ecotoxicology.

[13] Rüde H., Böhmer W., Müller M., Fliedner A., Ricking M., Teubner Diana., Schröter-Kermani C. (2013). Retrospective study of triclosan and methyl-triclosan residues in fish and suspended particulate matter: Results from the German Environmental Specimen Bank. Chemosphere.

[14] Adolfsson-Eric M., Pettersson M., Parkkonen J., Sturve J. (2002). Triclosan, a commonly used bactericide found in human milk and in the aquatic environment in Sweden. Chemosphere.

[15] Foran C.M., Bennett E.R., Benson W.H. (2000). Developmental evaluation of a potential non-steroidal estrogen: Triclosan. Mar. Environ. Res..

[16] Matsumura N., Ishibashi H., Hirano M., Nagao Y., Watanabe N., Shiratsuchi H., Kai T., Nishimura T., Kashiwagi A., Arizono K. (2005). Effects of nonylphenol and triclosan on production of plasma vitellogenin and testosterone in male South African clawed frogs (*Xenopus laevis*). Biol. Pharm. Bull..

[17] Dann A.B., Hontela A. (2011). Triclosan: Environmental exposure, toxicity and mechanisms of action. J. Appl. Toxicol..

[18] Meeker J.D., Cantonwine D.E., Rivera-González L.O., Ferguson K.K., Mukherjee B., Calafat A.M., Ye X., Toro A.D., Liza V., Crespo-Hernández N. (2013). Distribution, variability, and predictors of urinary concentrations of phenols and parabens among pregnant women in Puerto Rico. Environ. Sci. Technol..

[19] Calafat A.M., Ye X., Wong L.Y., Reidy J.A., Needham L.L. (2008). Urinary concentrations of triclosan in the US population: 2003–2004. Environ. Health Perspect..

[20] Gee R.H., Charles A., Taylor N., Darbre P.D. (2008). Oestrogenic and androgenic activity of triclosan in breast cancer cells. J. Appl. Toxicol..

[21] Prins G.S. (2008). Endocrine disruptors and prostate cancer risk. Endocr. Relat. Cancer.

[22] Kanetoshi A., Ogawa H., Katsura E., Kaneshima H. (1987). Chlorination of irgasan DP300 and formation of dioxins from its chlorinated derivatives. J. Chromatogr. A.

[23] Latch D.E., Packer J.L., Stender B.L., VanOverbeke J., Arnold W.A., McNeill K. (2005). Aqueous photochemistry of triclosan: Ormation of 2,4-dichlorophenol, 2,8-dichlorodibenzo-*p*-dioxin, and ligomerization products. Environ. Toxicol. Chem..

[24] Sires I., Oturan N., Oturan M.A., Rodriguez R., Garrido J.A., Brillas E. (2007). Electro-Fenton degradation of antimicrobials triclosan and triclocarban. Electrochim. Acta.

[25] Yang B., Ying G., Zhao J.L., Zhang L.J., Fang Y.X., Nghiem L.D. (2011). oxidation of triclosan by ferrate: Reaction kinetics, products identification and toxicity evaluation. J. Hazard. Mater..

[26] Munoz M., Pedro Z.M., Casas J.A., Rodriguez J.J. (2012). Triclosan breakdown by Fenton-like oxidation. Chem. Eng. J..

[27] Son H.S., Ko G., Zoh K.D. (2009). Kinetics and mechanism of photolysis and TiO_2_ photocatalysis of triclosan. J. Hazard. Mater..

[28] Latch D.E., Packer J.L., Arnold W.A., McNeill K. (2003). Photochemical conversion of triclosan to 2,8-dichlorodibenzo-*p*-dioxin in aqueoussolution. J. Photochem. Photobiol. A.

[29] Stamatics N., Antonopoulou M., Hela D., Konstantinou L. (2014). Photocatalytic degradation kinetics and mechanisms of antibacterial triclosan in aqueous TiO_2_ suspensions under simulated solar irradiation. J. Chem. Technol. Biotechnol..

[30] Kazushi A., James W. (2007). Photolytic degradation of triclosan in freshwater and seawater. Chemosphere.

[31] Sanchez-Prado L., Llompart M., Lores M., Fernandez-Alvarez M., Garcia J.C., Cela R. (2006). Further research on the photo-SPME of triclosan. Anal. Bioanal. Chem..

[32] Sun X.M., Zhang C.X., Zhao Y.Y., Bai J., Zhang Q.Z., Wang W.X. (2012). Atmospheric chemical reactions of 2,3,7,8-tetrachlorinated dibenzofuran initiated by an OH radical: Mechanism and kinetics study. Environ. Sci. Technol..

[33] Zhang C.X., Sun T.L., Sun X.M. (2011). Mechanism for OH-Initiated Degradation of 2,3,7,8-tetrachlorinated dibenzo-*p*-dioxins in the presence of O_2_ and NO/H_2_O. Environ. Sci. Technol..

[34] Yu J.C., Kwong T.Y., Luo Q., Cai Z. (2006). Photocatalytic oxidation of triclosan. Chemosphere.

[35] Zhang C.X., Zhao Y.Y., Bai J., Gong C., Sun X.M. (2012). Mechanism and kinetic study on the OH-initiated degradation of 2,3,7,8-tetrachlorinated dibenzofuran in atmosphere. Sci. Total Environ..

[36] Wong-Wah-Chung P., Rafqah S., Voyard G., Sarakha M. (2007). Photochemical behaviour of triclosan in aqueous solutions: Kinetic and analytical studies. J. Photochem. Photobiol. A.

[37] Guan B., Wan P. (1994). Photochemistry of dibenzo-1,4-dioxins: Intramolecular rearrangement-reduction through observable 2,2'-biphenylquinones. J. Photochem. Photobiol. A Chem..

[38] Kliegman S., Eustis S.N., Arnold W.A., McNeill K. (2013). Experimental and theoretical insights into the involvement of radicals in triclosan phototransformation. Environ. Sci. Technol..

[39] Zhao Y., Truhlar D.G. (2004). Hybrid meta density functional theory methods for thermochemistry, thermochemical kinetics, and noncovalent interactions: The MPW1B95 and MPWB1K models and comparative assessments forhydrogen bonding and van der Waals interactions. J. Phys. Chem. A Chem..

[40] Sun X.M., Bai J., Zhao Y.Y., Zhang C., Wang Y., Hu J.T. (2011). Chemical mechanism and kinetics study on the ocimene ozonolysis reaction in atmosphere. Atmos. Environ..

[41] Baldridge M.S., Gordon R., Steckler R., Truhlar D.G. (1989). *Ab initio* reaction paths and direct dynamics calculations. J. Phys. Chem..

[42] Gonzalez-Lafont A., Truong T.N., Truhlar D.G. (1991). Interpolated variational transition-state theory: Practical methods for estimating variational transition-state properties and tunneling contributions to chemical reaction rates from electronic structure calculations. J. Chem. Phys..

[43] Liu Y.P., Lynch G.C., Truong T.N., Lu D.H., Truhlar D.G., Garrett B.C. (1993). Molecular modeling of the kinetic isotope effect for the (1,5)-sigmatropic rearrangement of *cis*-1,3-pentadiene. J. Am. Chem. Soc..

